# Circulating microRNAs differentiate nociceptive and nociplastic pain: An exploratory study

**DOI:** 10.1016/j.ynpai.2025.100191

**Published:** 2025-07-05

**Authors:** Hiroyuki Nishie, Hideki Nakatsuka, Kazunori Iwasa, Yuka Sakuta, Yuichiro Toda, Shigeru Mitani, Takeshi Nagasaka

**Affiliations:** a Department of Advanced Oncology, Kawasaki Medical School, Kurashiki-City, Okayama 701-0192, Japan; b Department of Anesthesiology and Intensive Care Medicine, Kawasaki Medical School Hospital, Kurashiki-City, Okayama 701-0192, Japan; c Department of Psychology, Graduate School of Sustainable System Sciences, Osaka Metropolitan University, Osaka-City, Osaka, Japan; d Department of Bone and Joint Surgery, Kawasaki Medical School Hospital, Kurashiki-City, Okayama 701-0192, Japan

**Keywords:** Circulating miRNAs, Nociplastic pain, Nociceptive pain, Biomarker, Pain classification

## Abstract

**Background:**

Nociceptive and nociplastic pain arise from distinct biological mechanisms, yet their differentiation remains clinically challenging. Circulating microRNAs (miRNAs) are promising candidates for objective, mechanism-based pain classification.

**Objective:**

To explore whether specific circulating miRNAs can differentiate nociceptive pain in patients with hip osteoarthritis (HO) from nociplastic pain in patients with chronic primary pain (CPP), and to assess their relationship with clinical and psychological outcomes.

**Methods:**

In this exploratory, single-center study, plasma samples were collected from patients with HO (n = 13), CPP (n = 11), and healthy controls (n = 7). Microarray screening identified candidate miRNAs, which were validated via real-time PCR. Pain intensity (NRS), disability (PDAS), quality of life (EQ-5D), and psychological factors (PCS, PSEQ, TSK-11, PHQ-9) were assessed. Classification accuracy was evaluated using decision tree modeling and ROC analysis.

**Results:**

*Let-7a*, *miR-26a*, and *miR-16* showed distinct expression profiles and contributed to a predictive model with strong performance (*R^2^* = 0.677; AUC > 0.94). *Let-7a* expression was associated with structural joint changes in HO but not subjective pain ratings. *MiR-26a* correlated with cognitive-affective pain traits in CPP, and *miR-16* decreased following CBT, suggesting a role in treatment-related neuroplasticity. *MiR-126* and *miR-146a* were linked to reductions in pain intensity post-surgery in the HO group. QOL improved in HO, while psychological factors remained prominent in CPP.

**Conclusions:**

This pilot study suggests that circulating miRNAs may aid in differentiating nociceptive and nociplastic pain mechanisms and tracking treatment effects. While preliminary, these findings support the potential utility of miRNA-based biomarkers in precision pain diagnostics and personalized management strategies.

## Highlights

• *Let-7a*, *miR-26a*, and *miR-16* differentiate nociceptive and nociplastic pain.
• *Let-7a* tracks joint degeneration, not subjective pain.
• *MiR-16* decrease after CBT reflects neuroplastic adaptation.
• Decision tree model achieved AUC > 0.94 using miRNA markers.
• Circulating miRNAs show diagnostic potential in pain classification.

## Introduction

Pain is classified as nociceptive, neuropathic, or nociplastic, each requiring distinct treatments (NSAIDs, antiepileptics, or antidepressants). Distinguishing nociceptive from nociplastic pain remains challenging, emphasizing the need for objective biomarkers (Cohen et al., 2021, Finnerup et al., 2015, Fitzcharles et al., 2021). Nociceptive pain refers to pain caused by actual or threatened damage to non-neural tissue and is due to the activation of nociceptors, typically seen in acute injury or inflammatory conditions (Fitzcharles et al., 2021). In contrast, nociplastic pain arises from altered nociception without clear evidence of actual or threatened tissue damage or disease of the somatosensory system, and is commonly observed in conditions like fibromyalgia or complex regional pain syndrome (CRPS) (Fitzcharles et al., 2021). Additionally, animal studies have demonstrated that using opioids in the absence of inflammatory pain can lead to psychological addiction, highlighting the risks of misclassification (Suzuki et al., 1999).

MicroRNAs (miRNAs) have emerged as promising candidates for this purpose. These short, non-coding RNAs (∼22 nucleotides) regulate gene expression by binding to complementary sequences in target RNAs, playing critical roles in biological processes like cell differentiation and homeostasis (Bartel, 2018). Encapsulated in extracellular vesicles, miRNAs demonstrate remarkable stability in body fluids such as blood, cerebrospinal fluid, and saliva, which, combined with their specificity, non-invasive detectability, and long half-life, makes them ideal for biomarker development (Valadi et al., 2007, Wang et al., 2016).

Research has highlighted miRNAs as potential biomarkers for pain. For instance, *miR-146a-5p*, *miR-145-5p*, and *miR-130b-3p* have been identified as predictive markers for chronic pain following knee osteoarthritis surgery (Giordano et al., 2020). Similarly, *miR-223* has been linked to complex regional pain syndrome (CRPS) (Reinhold et al., 2023). A systematic review also identified miRNAs, including *miR-151*, *miR-23*, *miR-126*, and *miR-223*, as being associated with various conditions such as fibromyalgia, CRPS, neuropathy, and osteoarthritis (Polli et al., 2020). Despite these advancements, there has been no systematic comparison of miRNA expression between nociceptive and nociplastic pain, nor have changes in miRNA levels before and after treatment been extensively studied.

This study was designed as a pilot study to evaluate the potential of circulating miRNAs as objective biomarkers for pain classification. While the sample size is limited, this preliminary investigation provides essential insights for future large-scale studies.

## Methods

### Study design and participants

This single-center study (Approval #3057–03) followed the Declaration of Helsinki. Between May 2018 and May 2022, participants were recruited into three groups: HO (total hip arthroplasty for osteoarthritis), CPP (cognitive-behavioral therapy for chronic primary pain), and Control (healthy subjects). Inclusion/exclusion criteria are detailed in Supplementary Methods. This study has been registered in the UMIN Clinical Trials Registry (UMIN study ID: 000032416).

### Questionnaire survey

Participants completed paper-based self-administered questionnaires to assess treatment outcomes.

Quality of Life (QOL) was measured using EQ-5D-5L (EuroQol, 1990), pain intensity by Numeric Rating Scale (NRS) (Jensen et al., 1999), and disability by Pain Disability Assessment Scale (PDAS) (Yamashiro et al., 2011), Psychological aspects were assessed using Patient Health Questionnaire-9 (PHQ-9, mental health) (Spitzer et al., 1999, Muramatsu et al., 2007); Pain Catastrophizing Scale (PCS, pain catastrophizing) (Osman et al., 2000), Tampa Scale for Kinesiophobia-11 (TSK-11) (Kikuchi et al., 2015), and Pain Self-Efficacy Questionnaire (PSEQ, self-efficacy) (Nicholas, 2007, Adachi et al., 2014).

CPP and HO group participants completed all questionnaires before treatment and again at the time of post-treatment blood collection. Control group participants completed the questionnaires once, within one week before or after blood collection.

Missing data were handled as follows: in the HO group, one participant had a missing item in the PDAS and one in the TSK-11 at baseline, both imputed using the group median. Another HO case had a missing baseline value in the PSEQ, also imputed with the median. In the CPP group, one participant had a missing pre-treatment PCS score, which was imputed similarly.

### Blood collection and plasma storage

Blood samples from the HO group were collected immediately before surgery and approximately six months postoperatively. For the CPP group, samples were obtained before cognitive behavioral therapy (CBT) initiation and six months thereafter. Control group samples were collected on the day of questionnaire completion. Blood samples were collected using Insepac II-D vacuum blood collection tubes (SMD750EK-Yellow-ST, Sekisui Medical Co., Ltd., Tokyo, Japan), which contain a gel separator and are commonly used for plasma collection in clinical and research settings. Although not specifically designed for miRNA stabilization, samples were processed promptly and stored at − 80 °C to preserve RNA integrity, in accordance with previous recommendations for miRNA analysis (Mitchell et al., 2008, Cheng et al., 2013). Within one hour of collection, whole blood was centrifuged at 3000 rpm (approximately 1200 × g) for 10 min at 4 °C. The resulting plasma was carefully aliquoted and subjected to a second centrifugation at 3400 rpm (approximately 2000 × g) for 10 min at 4 °C to remove residual cellular debris and platelets.

Plasma samples were initially divided and stored separately at − 30 °C and − 80 °C. Plasma samples stored at − 80 °C were primarily used for RNA extraction and miRNA analysis in this study. In cases where − 80 °C samples were unavailable, plasma aliquots stored at − 30 °C were used, provided that they had not undergone multiple freeze–thaw cycles. Previous studies have shown that circulating miRNAs are stable in frozen plasma, including at − 30 °C, for several weeks to months, and tolerate limited freeze–thaw events without significant degradation (Kroh et al., 2010, McDonald et al., 2011).

### RNA extraction and quality assessment

Total RNA, including small RNAs, was extracted from 200 µL of plasma using one of two protocols, depending on the downstream analysis. For microarray analysis, RNA was extracted using the 3D-Gene RNA extraction reagent for liquid samples (Toray Industries, Kamakura, Japan). For quantitative real-time PCR (qRT-PCR) validation, the mirVana™ PARIS Kit (Thermo Fisher Scientific, Waltham, MA, USA) was used according to the manufacturer’s instructions.

Total RNA (including small RNAs) was extracted from plasma for microarray profiling. RNA Integrity Number (RIN) was measured on these total RNA extracts as part of Toray Industries’ internal quality control. However, the interpretation of RIN in plasma RNA is limited due to the naturally fragmented and ribosome-deficient state of extracellular RNAs.

RNA concentration and integrity were assessed only for microarray samples using Toray Industries’ internal quality control platform as part of their microarray service. RNA integrity was evaluated in eight representative plasma RNA samples, yielding RIN values ranging from 2.10 to 4.40. This range is consistent with expectations for plasma-derived RNA, which typically lacks intact ribosomal RNA and is inherently fragmented due to its extracellular origin and susceptibility to nuclease activity (McDonald et al., 2011, El-Khoury et al., 2016). All tested samples met the predefined threshold of RIN ≥ 2.0, which was used to confirm consistency and quality for microarray-based analysis.

However, because RIN primarily reflects ribosomal RNA integrity, it is not considered a suitable quality control metric for qRT-PCR analysis of plasma miRNAs, which are small, extracellular, and largely non-ribosomal. Consequently, RIN was not assessed for qRT-PCR samples. Instead, RNA quality was assured by two complementary measures: (1) the use of a commercially validated miRNA-specific extraction kit, and (2) consistent amplification of an exogenous spike-in control (cel-miR-39), following best practices described in previous studies (McDonald et al., 2011, McAlexander et al., 2013).

These methods provide appropriate quality assurance tailored to the unique properties of plasma miRNAs. Prior research has demonstrated that reproducible plasma miRNA analysis can be achieved even in the absence of intact ribosomal RNA, as long as validated extraction protocols and proper normalization controls are applied (Cheng et al., 2013, McDonald et al., 2011, McAlexander et al., 2013). This is further supported by Garcia-Elias et al., who demonstrated that meaningful microarray-based quantification of plasma miRNAs can be performed despite the lack of detectable 18S and 28S rRNA due to the naturally fragmented and ribosome-deficient nature of circulating plasma RNA, as also illustrated by the absence of 18S and 28S peaks (Garcia-Elias et al., 2017).

### Microarray analysis for miRNA expression profiling

Microarray analysis was conducted using plasma RNA samples from two HO cases and two CPP cases who completed treatment and demonstrated clear improvement in questionnaire-based symptom scores. Following quality assessment, RNA samples were labeled using the 3D-Gene miRNA labeling kit (Toray) and hybridized to the Human miRNA Oligo Chip (Toray), with probe annotations based on miRBase. Raw fluorescence intensities were processed using 3D-Gene Extraction Software (Toray). For background correction, each spot's signal intensity was adjusted by subtracting the mean intensity of blank spots calculated within the 95 % confidence interval. Only signals exceeding two standard deviations above background were retained. Global normalization to a median signal value of 25 per array was then performed to minimize inter-array variability.

All normalized signal intensity values for individual samples are presented in Supplementary Table 1, confirming that no sample pooling occurred and that all analyses were conducted at the individual level.

### Selection criteria for candidate miRNAs

Candidate miRNAs for distinguishing nociceptive from nociplastic pain were selected based on the following criteria:

1) The value of global normalization after treatment minus the value of global normalization before treatment in the HO group (Δ HO global normalization) > 10, which ensures the selection of miRNAs that significantly increase following surgery and are potentially linked to nociceptive pain resolution.

2) −100 < the value of global normalization after treatment minus the value of global normalization before treatment in the CPP group (Δ CPP global normalization) < 0, ensuring that the selected miRNAs were not upregulated in the CPP group, thereby minimizing the influence of nociplastic pain mechanisms.

3) Pre-treatment HO/CPP global normalization ratio < 0.7, indicating that these miRNAs had lower baseline expression in HO patients than in CPP patients, reinforcing their specificity to nociceptive pain.

The biological rationale for these thresholds is detailed in the Supplementary Methods, prioritizing miRNAs associated with peripheral nociceptive resolution processes. Hierarchical cluster analysis based on Δ HO and Δ CPP values was also performed to identify potential biomarkers.

### Quantitative real-time PCR (qRT-PCR) analysis

*Synthetic Caenorhabditis elegans miR-39-3p* (*cel-miR-39*; Qiagen, final concentration: 10 pM) was spiked into each sample exclusively as an exogenous control for the real-time PCR experiments. It was not used in any part of the microarray analysis. Reverse transcription was performed using the TaqMan™ MicroRNA Reverse Transcription Kit (Thermo Fisher Scientific) in a 15 µL reaction volume containing 0.15 µL of dNTPs, 1 µL of MultiScribe™ reverse transcriptase, 1.5 µL of reverse transcription buffer, 0.19 µL of RNase inhibitor, 3 µL of miRNA-specific primers, and the extracted RNA.

qRT-PCR was conducted using the StepOne® Plus Real-Time PCR System (Thermo Fisher Scientific) with TaqMan™ Fast Advanced Master Mix and miRNA-specific TaqMan™ assays (Thermo Fisher Scientific, catalog no. 4427975). Each 20 µL reaction included 10 µL of master mix, 1.33 µL of cDNA, and 1 µL of the specific miRNA assay. Reactions were run under the following cycling conditions: 95 °C for 20 s, followed by 40 cycles of 95 °C for 1 s and 60 °C for 20 s.

miRNA expression levels were quantified using the 2^−ΔΔCt method, where ΔCt = Ct(target miRNA) − Ct(*cel-miR-39*). Expression data were normalized to a reference control sample from the healthy control group for relative quantification.

Primers for miRNA-specific primer sequences were purchased from Thermo Fisher. The primers (catalog No. 4427975) were as follows: *Cel-miR-39-3p*, *has-miR-16-5p* (*miR-16*), *hsa-miR-26a-5p* (*miR-26a*), *hsa-let-7a-5p* (*let-7a*), *has-let-7d-5p* (*let-7d*), *has-miR-103a-3p* (*miR-103a*), *has-miR-223-3p* (*miR-223*), *has-miR146a-5p* (*miR-146a*), *has-miR-126-3p* (*miR-126*), and *has-miR21-5p* (*miR-21*).

All real-time PCR analyses were conducted by investigators blinded to group assignment and clinical outcomes, including pre/post-treatment status and QOL scores.

### Statistical analysis

The primary objective was to identify circulating microRNAs (miRNAs) that exhibit differential expression before and after treatment in the CPP and HO groups and to assess their potential for distinguishing nociceptive and nociplastic pain.

Sample size was determined using G*Power 3.1 software with α = 0.05, power = 0.80, and an assumed large effect size (Cohen’s d = 0.8), yielding a minimum requirement of 12 participants per group. (Faul et al., 2007, Dieppe and Lohmander, 2005) To account for possible dropouts or sample loss, we recruited 15 participants per group. The control group was limited to 10 participants due to budgetary constraints.

All analyses were performed using JMP Pro version 17.0 (SAS Institute, Cary, NC, USA). From an initial dataset of 2,632 miRNAs, 1,189 met quality control thresholds and were retained for analysis. Background correction was applied by estimating the mean intensity of blank spots within a 95 % confidence interval, and global median normalization was applied to rescale the median intensity per array to 25.

Normality of distributions was tested using the Shapiro–Wilk test. Given the small sample sizes and non-normal distributions, nonparametric tests were employed throughout. Between-group comparisons were conducted using the Steel–Dwass test, and within-group pre–post comparisons used the Wilcoxon signed-rank test. Associations between miRNA expression and clinical variables were examined using Spearman’s rank correlation coefficient.

To correct for multiple comparisons in the high-dimensional miRNA dataset, p-values were adjusted using the Benjamini–Hochberg false discovery rate (FDR) method. A two-tailed FDR-adjusted *P* < 0.05 was considered statistically significant.

We performed recursive partitioning analysis (decision tree modeling) using the Partition platform in JMP Pro, with likelihood-ratio chi-square (*G^2^*) as the split criterion to assess the diagnostic potential of selected miRNAs. Model performance was evaluated using the coefficient of determination (*R^2^*) and area under the receiver operating characteristic curve (AUC), calculated from internal ROC curves generated within JMP. No external validation was performed due to the exploratory nature and limited sample size. Rank-biserial correlation was also reported as an effect size measure for pairwise comparisons.

Unless otherwise stated, data are presented as median (interquartile range, IQR) or mean (95 % confidence interval, CI).

## Results

### Participant recruitment and characteristics

**Fig. 1:**
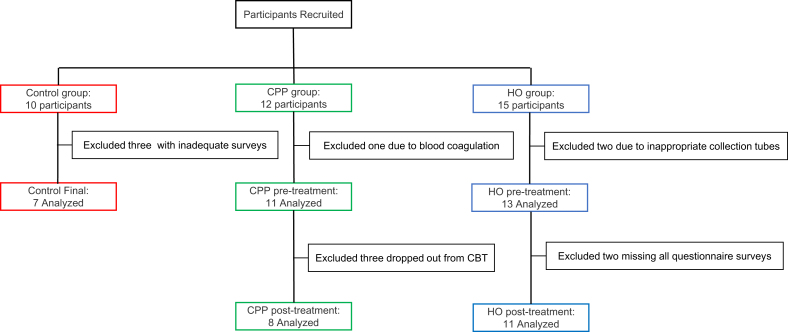
Patient recruitment and analysis flow.

Fig. 1 summarizes the recruitment and selection process of study participants. During the study period, 12 participants were recruited for the CPP group, 15 for the HO group, and 10 for the Control group.

Although the target sample size for the CPP group was 15, only 12 cases were enrolled. One participant was excluded due to blood sample coagulation, resulting in 11 participants for pre-treatment analysis. Additionally, three participants discontinued CBT and were excluded from the post-treatment analysis.

In the HO group, two pre-treatment blood samples were collected using incorrect tubes, and two participants lacked both post-treatment blood samples and questionnaire data. As a result, 13 HO participants were included in the pre-treatment analysis and 11 in the post-treatment analysis.

In the Control group, two participants were excluded due to unusually low EQ-5D-5L scores, and one was excluded based on a combination of a high NRS score (Bartel, 2018) and a markedly low PSEQ score. Consequently, seven Control participants were included in the final analysis.

In summary, the final dataset included 11 CPP participants, 13 HO participants, and 7 Controls. Participant characteristics are presented in **Supplementary Table 2**, and pain site distribution and treatment outcomes for the CPP group are detailed in Supplementary Table 3**.**

### QOL and pain-related measures

HO patients significantly improved EQ-5D, NRS, and PDAS, while CPP had non-significant changes (Table 1). Pain catastrophizing decreased in both groups, with CPP patients showing significant improvements in self-efficacy and kinesiophobia (*P* = 0.047 and 0.03, respectively). Depression severity remained unchanged. Overall, surgery (HO) led to improvements in pain, disability, and QOL, while CBT had a modest effect in the CPP group.

### MiRNA candidates identified via microarray analysis

Microarray analysis identified 25 miRNAs that met predefined selection criteria (Table 2), representing potential markers of nociceptive pain in the HO group and excluding those influenced by nociplastic mechanisms in the CPP group.

**Table 1:** Results of Questionnaire Surveys.

	**Control (n = 7)**	**CPP**			***P*-value**	**HO**			***P*-value**
		**Pre-treatment (n = 11)**	**Post-treatment (n = 8)**	**Δ (n = 8)**		**Pre-treatment (n = 13)**	**Post-treatment (n = 11)**	**Δ (n = 11)**	
EQ-5D [range: 0–1], Mean (95 %CI)	1 (1−1)	0.6 (0.5−0.7))	0.8 (0.6−0.9)	0.2 (0−0.4)	0.1	0.6 (0.5−0.7)	0.9 (0.8−0.9)	0.3 (0.1−0.4)	**0.005**

NRS [range: 0–10], Mean (95 %CI)									
Maximum	1.1 (−0.1 to 2.4)	7.0 (5.7–8.3)	6.1 (3.8–8.4)	−0.9（−2.9 to 1.2）	0.5	5.8 (4.6–6.9)	1.4 (0.3–2.5)	−4.6 (−6.3 to −2.9)	**0.002**
Minimum	0 (0)	2.8 (0.7–4.4)	2.6 (−0.1 to 5.4)	−0.3 (−1.7 to 1.2)	1.0	2.9 (1.5–4.4)	0.5 (−0.1 to 1.0)	−2.6 (−4.5 to −08)	**0.016**
Average	0.3 (−0.4 to 1.0)	5.3 (3.8–6.8)	4.8 (2.6–6.9)	−0.6 (−2.0 to 0.8)	0.4	4.7 (3.7–5.7)	1.0 (0.2–1.8)	−3.9 (−5.3 to −2.5)	**0.001**
Current	0.1 (−0.2 to 0.5)	5.7 (4.2–7.3)	3.8 (0.7–6.8)	−2.0 (−4.4 to 0.4)	0.1	4.5 (3.4–5.5)	0.7 (−0.1 to 1.53	−3.8 (−5.3 to −2.3)	**0.002**

PDAS [range: 0–60], Mean (95 %CI)	0.1 (−0.2 to 0.5)	19.6 (12.9–26.2)	13.5 (2.7–24.4)	−7.1 (−15.3 to 1.1)	0.1	25.3 (20.9–29.8)	9.2 (4.0–5.4)	−15.5 (−22.3 to −8.6)	**0.003**

PHQ-9 [range: 0–27]. Mean (95 %CI)	0.3 (−0.4 to 11.0)	10.2 (7.4–13.0)	6.0 (2.5–9.5)	−3.8 (−8.6 to 1.1)	0.2	4.5 (1.6–7.5)	2.23(0–4.5)	−1.4 (−4.1 to 1.3)	0.4

PCS, Mean (95 %CI)									
Overall [range: 0–52]	0 (0–0)	35.1 (28.9–41.3)	21.1 (7.5–34.7)	−13.4 (−25.4 to −1.4)	**0.008**	21.8 (13.7–29.9)	6.0 (1.8–10.2)	−14.3 (−23.7 to −4.9)	**0.006**
Rumination [range: 0–20]	0 (0–0)	15.6 (13.1–18.2)	10.6 (4.5–16.7)	−4.6 (−10.0 to 0.7)	0.1	12.0 (9.0–15.0)	3.4 (0.9–5.9)	−7.8 (−12.3 to −3.3)	**0.005**
Helplessness [range: 0–20]	0 (0–0)	12.1 (9.1–15.1)	6.1 (1.7–10.6)	−5.5 (−10.0 to −1.1)	**0.017**	6.2 (2.6–9.9)	1.1 (−0.1 to 1.2)	−4.6 (−8.7 to −0.6)	**0.03**
Magnification [range: 0–12]	0 (0–0)	7.6 (5.9–9.2)	4.4 (1.1–7.7)	−3.3 (−5.8 to −0.7)	**0.03**	3.6 (1.7–5.6)	1.6 (0.7–2.41	−1.8 (−3.8 to 0.1)	0.06

TSK-11 [range: 11–44], Mean (95 %CI)	11.4 (10.4–12.5)	29.1 (26.1–32.1)	22.1 (17.3–27.0)	−7.0 (−14.9 to 0.9)	**0.047**	25.2 (22.1–28.2)	21.0 (17.5–24.5)	−3.6 (−7.2 to −0.1)	0.07

PSEQ [range: 0–60], Mean (95 %CI)	51.7 (3768–65.9)	24.1 (15.1–32.5)	33.6 (22.3–44.9)	14.0 (2.8–25.2)	**0.03**	37.0 (30.0–44.0)	40.3 (32.6–48.9)	4.6 (−5.1 to 14.2)	0.5

Δ values were the values obtained from subtracting the pre-treatment scores from post-treatment.

*P*-values were evaluated using the Wilcoxon Signed Rank Test.

**Table 2:** Candidate miRNAs identified by microarray analysis.

Order	miRNA	miRBase Accession Number	Pre_HO_mean/Pre_CPP_mean < 0.7	10 < Δ HO global normalization	−100 < Δ CPP global normalization < 0
**1**	***hsa-miR-16-5p***	**MIMAT0000069**	**0.30846135**	**65.4621385**	**−55.9684785**
2	*hsa-miR-320e*	MIMAT0015072	0.65743498	57.4755425	−9.729112
**3**	***hsa-let-7a-5p***	**MIMAT0000062**	**0.282983665**	**56.48857**	**−6.5375995**
**4**	***hsa-miR-26a-5p***	**MIMAT0000082**	**0.38195271**	**41.259165**	**−14.9463035**
**5**	***hsa-let-7d-5p***	**MIMAT0000065**	**0.289671993**	**41.0947605**	**−12.8727615**
**6**	***hsa-miR-223-3p***	**MIMAT0000280**	**0.459143593**	**33.9152385**	**−79.870273**
7	*hsa-miR-23a-3p*	MIMAT0000078	0.682647055	32.10724	−4.705914
8	*hsa-miR-614*	MIMAT0003282	0.647765936	28.429501	−23.488508
**9**	***hsa-miR-103a-3p***	**MIMAT0000101**	**0.38938391**	**27.9834375**	**−12.0402905**
10	*hsa-miR-92b-3p*	MIMAT0003218	0.588736951	25.3022005	−16.1860505
11	*hsa-miR-106a-5p*	MIMAT0000103	0.38212634	23.866509	−2.9233
12	*hsa-let-7f-5p*	MIMAT0000067	0.180966444	23.7144765	−14.88124
13	*hsa-miR-20a-5p*	MIMAT0000075	0.347556794	22.17461	−5.6716035
**14**	***hsa-miR-21-5p***	**MIMAT0000076**	**0.613098476**	**20.8112715**	**−4.6412955**
15	*hsa-miR-17-5p*	MIMAT0000070	0.369398712	19.982459	−8.384574
16	*hsa-miR-20b-5p*	MIMAT0001413	0.319838872	18.6637025	−2.7079695
**17**	***hsa-miR-126-3p***	**MIMAT0000445**	**0.415322564**	**16.736257**	**−27.2786605**
18	*hsa-miR-107*	MIMAT0000104	0.375100328	15.266438	−13.338976
19	*hsa-miR-3680-3p*	MIMAT0018107	0.609013966	15.0309325	−4.8702155
20	*hsa-let-7e-5p*	MIMAT0000066	0.256149136	14.713242	−3.8552805
**21**	***hsa-miR-146a-5p***	**MIMAT0000449**	**0.663682119**	**12.365162**	**−0.5431425**
22	*hsa-miR-12117*	MIMAT0049011	0.637711133	12.160739	−0.050295
23	*hsa-miR-30b-5p*	MIMAT0000420	0.399159519	11.343624	−5.91375
24	*hsa-miR-26b-5p*	MIMAT0000083	0.114167274	10.83154	−8.03152
25	*hsa-miR-191-5p*	MIMAT0000440	0.615280291	10.0229465	−2.230807

Δ HO global normalization denotes the value of global normalization after treatment minus the value of global normalization before treatment in the HO group.

Δ CPP global normalization denotes the value of global normalization after treatment minus the value of global normalization before treatment in the CPP group.

To further refine the candidate selection, we categorized all 1,189 detected miRNAs into four expression-based quadrants according to their change direction in the HO and CPP groups (Supplementary Table 4). This 2 × 2 matrix based on Δ global normalization (ΔHO vs. ΔCPP) revealed that the majority of miRNAs (78.6 %) fell within the quadrant where both ΔHO and ΔCPP were less than zero. In contrast, only 7.1 % of miRNAs were uniquely upregulated in the HO group while being downregulated in CPP (ΔHO > 0, ΔCPP < 0)—a pattern potentially reflective of nociceptive-specific resolution mechanisms.

Four representative miRNAs from the quadrant characterized by ΔHO > 0 and ΔCPP < 0 were identified based on their high ΔHO global normalization values and known biological relevance in pain and inflammation pathways. These included *miR-16, let-7a*, *let-7d*, and *miR-26a*, clustered in unsupervised analyses (Supplementary Table 5**, Supplementary** Fig. 1).

In parallel, a literature-driven approach guided by Polli et al. (Polli et al., 2020) was employed to include *miR-223*; *miR-103a*, *miR-21*, *miR-126*, and *miR-146a*, each previously reported as highly relevant to chronic pain phenotypes.

As a reference, Supplementary Table 6 presents illustrative examples of representative miRNAs from each ΔHO/ΔCPP quadrant, annotated with fold changes and relevant biological functions. Although not directly used in the selection process, this table helps contextualize the quadrant-based framework and highlights the biological heterogeneity across expression patterns.

Combining expression quadrant analysis, statistical ranking, and literature support, this integrative strategy yielded a focused list of 25 candidate miRNAs. Among these, nine miRNAs were selected for real-time PCR experimental validation, based on their differential expression patterns and relevance to nociceptive or nociplastic pain mechanisms.

### qRT-PCR to confirm reproducibility

**Fig. 2:**
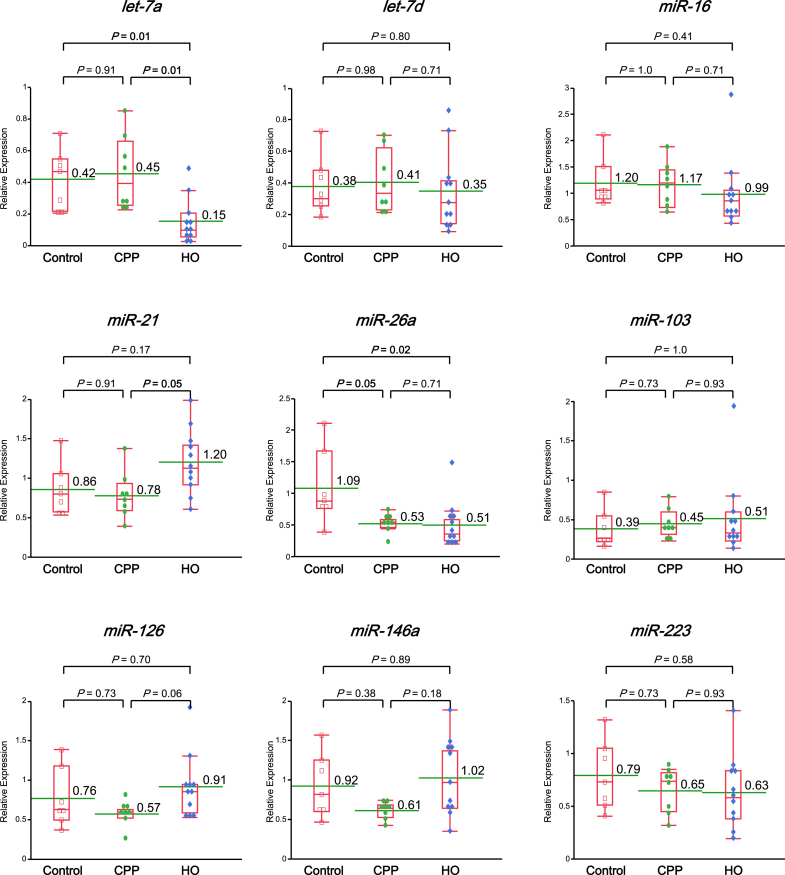
MiRNA expression levels in Control, CPP, and HO groups. Relative expression levels of selected miRNAs (*let-7a*, *miR-16*, *miR-26a*, etc.) in Control, CPP, and HO groups. Relative expression was calculated using the 2^−ΔΔCt method, normalized to the spike-in control *cel-miR-39*. The horizontal line within each red box represents the median; the limits of each box correspond to the interquartile ranges; the whiskers indicate the maximum and minimum values; and the green horizontal bar within each box depicts the mean value. The numerical values above the green horizontal bars indicate the means. Statistical comparisons were performed using the Steel-Dwass method. *P*-values are indicated for each comparison. Statistically significant differences (*P* < 0.05) are highlighted. (For interpretation of the references to colour in this figure legend, the reader is referred to the web version of this article.)

To compare pre-treatment miRNA expression among groups (Fig. 2), we found that *let-7a* was significantly downregulated in the HO group compared to the Control group (HO vs. Control, *P* = 0.01; CPP vs. HO, *P* = 0.01). Similarly, *miR-26a* expression was significantly lower in both the CPP and HO groups compared to Controls (HO vs. Control, *P* = 0.02; CPP vs. Control, *P* = 0.05). Additionally, miR-21 expression differed significantly between the CPP and HO groups (*P* = 0.05).

**Fig. 3:**
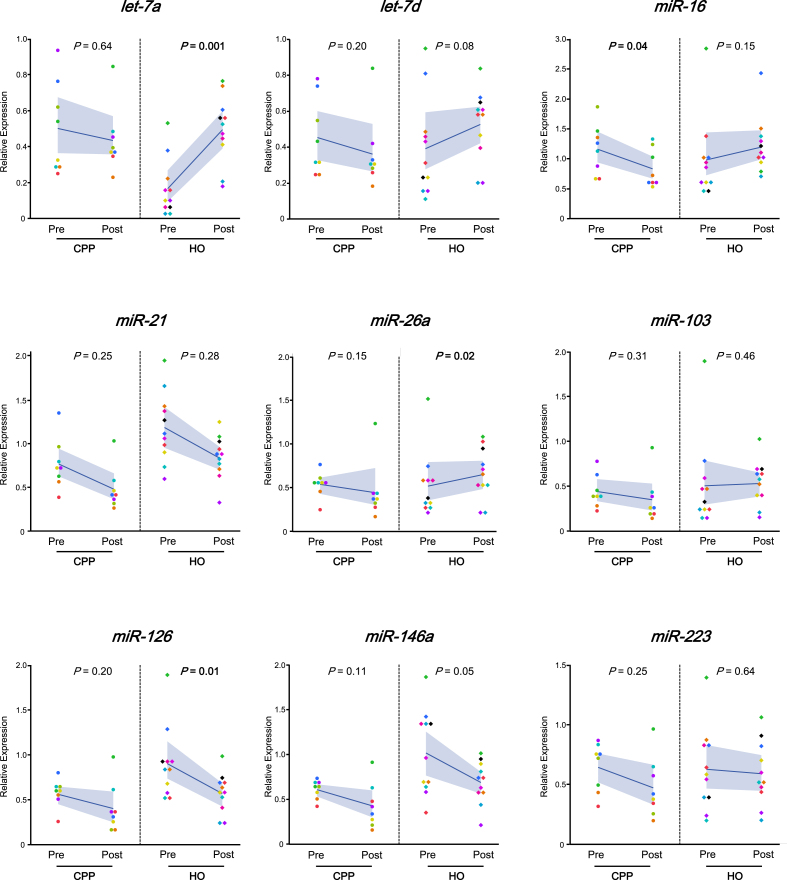
Changes in circulating miRNA expression before and after treatment in CPP and HO groups. Each plot shows individual expression values of nine selected microRNAs (l*et-7a*, *let-7d*, *miR-16*, *miR-21*, *miR-26a*, *miR-103*, *miR-126, miR-146a*, and *miR-223*) measured before (Pre) and after (Post) treatment in patients with chronic primary pain (CPP, n = 8) and hip osteoarthritis (HO, n = 11). Only patients with paired blood samples collected both before and after treatment were included in the analysis shown here. Relative expression was calculated using the 2^−ΔΔCt method, normalized to the spike-in control *cel-miR-39*. The blue line represents the mean value, and the gray shaded area indicates the 95 % confidence interval (CI). Statistical comparisons were performed using the Wilcoxon signed-rank test. *P* values < 0.05 are shown in bold. Note: The number of cases shown here differs from those in Supplementary Table 4, which includes all available samples regardless of pairing. (For interpretation of the references to colour in this figure legend, the reader is referred to the web version of this article.)

Regarding changes in expression before and after treatment (Fig. 3), *let-7a* expression significantly increased post-surgery in the HO group (*P* = 0.001). Conversely, *miR-26a* (*P* = 0.02) and *miR-126* (*P* = 0.01) were significantly downregulated after surgery. In the CPP group, only *miR-16* showed a significant decrease following CBT (*P* = 0.04); no other miRNAs showed significant changes.

Mean expression values and 95 %CIs for all nine miRNAs before and after treatment are shown in Supplementary Table 7.

### Distinct miRNA-clinical associations in CPP and HO

Spearman’s rank correlation coefficient (*ρ*) was used to evaluate relationships between changes in miRNA expression and clinical variables (Supplementary Table 8).

In the CPP group, *miR-26a* showed a negative correlation with the PCS rumination subscale (*ρ* =  − 0.708, *P* = 0.050), suggesting its involvement in cognitive-affective pain processing. No other correlations reached statistical significance.

In the HO group, *miR-126*, *miR-146a*, *let-7d*, and *miR-21* correlated with reductions in pain intensity (NRS scores). *Let-7d* and *miR-21* were associated with minimum pain scores, while *miR-126* and *miR-146a* correlated with broader NRS measures. However, after FDR correction, no significant correlations were observed with QOL measures (EQ-5D, PCS, PSEQ).

These findings suggest that *miR-26a* may modulate cognitive-affective pain processing in CPP, while *miR-126* and *miR-146a* contribute to pain relief mechanisms in HO, reflecting distinct underlying pain mechanisms.

### Partition analysis to identify key miRNAs for pain classification

To assess whether pre-treatment miRNA expression could predict pain type (nociceptive, nociplastic, or no pain), partition analysis (decision tree modeling) was performed.

*Let-7a* was identified as the primary split variable, demonstrating strong discriminatory ability between nociceptive (HO) and nociplastic (CPP) pain. Notably, although *let-7a* was significantly upregulated postoperatively in HO participants, it was not correlated with pain intensity measures. This suggests that *let-7a* may reflect structural joint changes rather than subjective pain, making it a suitable marker for initial classification.

**Fig. 4:**
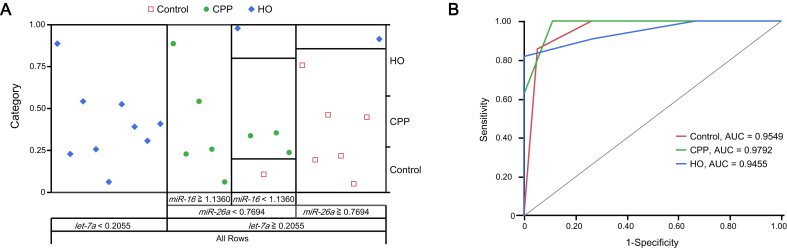
Decision tree model for pain classification. (A) Decision tree model classifying patients into Control, CPP, and HO groups based on miRNA expression levels. The model first splits at *let-7a* expression level, followed by *miR-26a* and *miR-16*, to refine classification. (B) Receiver Operating Characteristic (ROC) curves for miRNA-based CPP, HO, and control group classification. The model achieved excellent classification performance, with area under the curve (AUC) values exceeding 0.90, a threshold indicative of highly discriminative diagnostic models.

The partition model demonstrated strong performance (*R^2^* = 0.677) and used three hierarchical splits to classify 26 samples (Fig. 4**A**). The first split was based on *let-7a* expression (threshold: 0.2055); samples below this level (n = 9) were perfectly classified (*G^2^* = 0). For samples ≥ 0.2055 (n = 17), a second split was made using *miR-26a* (threshold: 0.7694). Among these, those with *miR-26a* < 0.7694 (n = 10) were further split by *miR-16* at 1.1360. Samples with *miR-16* ≥ 1.1360 were fully classified (*G^2^* = 0), while those below this threshold retained some variability (*G^2^* = 9.50). Samples with *miR-26a* ≥ 0.7694 (n = 7) showed lower classification confidence (*G^2^* = 5.74).

Model performance was evaluated using ROC analysis, yielding high AUC values for all groups: 0.9549 (Control), 0.9792 (CPP), and 0.9455 (HO) (Fig. 4**B**). These results support the use of *let-7a*, *miR-26a*, and *miR-16* as key biomarkers for pain classification.

## Discussion

This exploratory study identified distinct circulating miRNA profiles associated with nociceptive pain (HO group) and nociplastic pain (CPP group), supporting their potential as objective biomarkers for pain classification. Among the candidate miRNAs, *let-7a*, *miR-26a*, and *miR-16* demonstrated notable discriminative and predictive capabilities, suggesting involvement in structural pathology, neuroplasticity, and therapeutic response. These findings offer preliminary support for the use of miRNA signatures to complement subjective pain assessment and facilitate personalized pain management.

To further delineate the biological relevance of circulating miRNAs in relation to nociceptive and nociplastic mechanisms, we categorized all 1,189 miRNAs into four quadrants based on the direction of their expression change following treatment in the HO and CPP groups (Supplementary Table 4).

This 2 × 2 matrix enabled a stratified interpretation of miRNA behavior as follows: (A) ΔHO > 0 / ΔCPP < 0 — candidate nociceptive resolution markers, as these miRNAs were upregulated in the HO group but downregulated or unchanged in the CPP group; (B) ΔHO < 0 / ΔCPP < 0 — miRNAs downregulated across both groups, potentially reflecting shared treatment-related immune modulation; (C) ΔHO > 0 / ΔCPP > 0 — miRNAs upregulated in both groups, likely representing non-specific recovery or stress-related responses; and (D) ΔHO < 0 / ΔCPP > 0 — miRNAs with increased expression specific to the CPP group, which may be involved in maladaptive neuroplasticity or nociplastic pain mechanisms. This classification framework, combined with expression magnitude ranking and literature review, facilitated the selection of biologically relevant miRNAs for further validation (Supplementary Table 6).

In the HO group, circulating *let-7a* was significantly downregulated prior to surgery but showed marked upregulation postoperatively. This pattern suggests that *let-7a* plays a role in resolving chronic inflammation following removal of the degenerated joint. The *let-7* family is known to modulate neuroinflammation through Toll-like receptor 7 (TLR7) signaling and cytokine regulation, including IL-6 and TNF-α suppression (Fiebich et al., 2018, Ma et al., 2021, Song and Lee, 2015). *Let-7a* also regulates the activity of ASK1 (apoptosis signal-regulating kinase 1), a MAP kinase kinase kinase that mediates stress and inflammatory signaling in neurons and glial cells. Through its modulation of ASK1 in microglia, *let-7a* influences the balance between pro- and anti-inflammatory cytokines, thereby contributing to neuroprotection and pain resolution (Song and Lee, 2015). In musculoskeletal conditions, *let-7a* additionally regulates osteoblast differentiation and bone metabolism, though with bidirectional effects depending on the target (Alrashed et al., 2022, Ma et al., 2019). In our study, *let-7a* expression was not correlated with subjective pain or QOL metrics, suggesting that its recovery post-surgery primarily reflects resolution of mechanical stress and structural degeneration, rather than pain perception itself. Thus, *let-7a* may serve as a biomarker of joint pathology rather than a direct pain indicator.

In contrast, *miR-26a* expression was suppressed in both CPP and HO groups prior to treatment, and remained low postoperatively in HO. Previous studies have linked *miR-26a* to neuroplasticity and regulation of neuroinflammatory cytokines such as IL-6 and TNF-α (Kumar et al., 2015). The similar suppression of *miR-26a* in both patient groups suggests that it may reflect broader alterations in central pain modulation rather than structural damage alone. Notably, *miR-26a* correlated with cognitive-affective traits (e.g., rumination) in the CPP group, reinforcing its role in psychological dimensions of pain processing. These findings position *miR-26a* as a candidate marker of affective-cognitive dysregulation in chronic pain.

Circulating *miR-16* emerged as another relevant biomarker, particularly in nociplastic pain. Widely studied in cancer biology, *miR-16* also regulates inflammatory and apoptotic pathways (Ghafouri-Fard et al., 2022). In our study, its expression decreased significantly following CBT in the CPP group, possibly reflecting adaptive neuroplasticity induced by psychological intervention. Nociplastic pain is characterized by central sensitization—persistent amplification of pain without peripheral tissue damage (Woolf, 2011). While canonical markers such as BDNF, S100B, and GFAP reflect glial activation and central pain facilitation (Woolf, 2011, Loggia et al., 2015); *miR-16* may capture a more dynamic component of neural plasticity and recovery. Its decline following CBT suggests responsiveness to non-pharmacological therapy and potential utility for monitoring treatment effects in centrally-mediated pain.

In contrast, several miRNAs showed decreased expression levels after treatment, which may indicate resolution of pathological states such as inflammation or central sensitization. Circulating *miR-16*, which plays roles in apoptotic and inflammatory signaling pathways, was significantly reduced in the CPP group after CBT, possibly reflecting reduced central sensitization. This change, while not directly correlated with VAS pain scores, may reflect a broader therapeutic response including affective and cognitive domains. Similarly, *miR-126* and *miR-146a*, involved in immune signaling and vascular inflammation, decreased post-treatment in the HO group. These miRNAs have been previously implicated in nociceptive mechanisms, and their reduction may indicate structural or inflammatory recovery following surgery. Taken together, both upregulation and downregulation of specific miRNAs appear relevant in reflecting pathophysiological changes and therapeutic effects in chronic pain, emphasizing their potential utility as dynamic treatment-response biomarkers.

Furthermore, *let-7a*, *miR-26a*, and *miR-16* have been implicated in other chronic pain conditions such as fibromyalgia, neuropathic pain, and osteoarthritis, as summarized in Supplementary Table 9, further supporting their relevance as broadly applicable pain-related biomarkers.

In the HO group, pain relief after surgery was associated with changes in additional miRNAs, including *miR-126*, *miR-146a*, *let-7d*, and *miR-21*. These miRNAs have established roles in vascular function, immune signaling, and inflammation (Wang et al., 2016, Jensen et al., 1999). Their correlations with pain intensity suggest that they may mediate structural or inflammatory pathways contributing to nociceptive pain resolution. Importantly, the CPP group showed limited correlation between miRNA changes and pain intensity, but stronger links to psychological traits such as catastrophizing and kinesiophobia. This divergence supports the biological distinction between nociceptive and nociplastic pain and emphasizes the need for mechanism-specific diagnostics and interventions.

Our findings are consistent with previous reports of circulating miRNA alterations in chronic pain conditions, particularly nociplastic syndromes such as fibromyalgia. Prior studies have identified circulating miRNA panels that distinguish fibromyalgia from other disorders, with some overlap in miRNAs observed in our study (Giordano et al., 2020, Polli et al., 2020). These findings reinforce the concept that circulating miRNAs reflect central sensitization and cognitive-affective dysregulation, hallmark features of nociplastic pain.

Our decision tree model, based on pre-treatment miRNA expression, achieved high classification accuracy (AUC > 0.94), with *let-7a*, *miR-26a*, and *miR-16* emerging as key discriminatory markers. Although promising, these results must be interpreted cautiously given the small sample size and risk of overfitting. Nonetheless, they underscore the potential of integrating miRNA-based diagnostics with machine learning to improve pain classification.

### Limitations and future directions

This study has several limitations. The small sample size, particularly in the CPP and Control groups, limits statistical power and generalizability. Despite FDR correction, the high-dimensional nature of microarray data raises the risk of false positives. The use of *cel-miR-39* as a normalization control, while common in biofluid miRNA studies, lacks an endogenous stability reference and may introduce variability. Furthermore, the absence of neuropathic pain—a major clinical pain subtype—limits the scope of the classification model.

Circulating miRNA studies are further challenged by pre-analytical variables such as hemolysis, sample collection and storage conditions, RNA yield, and platform-specific differences. These issues affect reproducibility and comparability across studies and remain a central limitation of miRNA biomarker research (McDonald et al., 2011). Nonetheless, we addressed these limitations by applying a dual approach to RNA quality control. While RNA integrity was verified for microarray samples using RIN values (RIN ≥ 2.0), this metric is not suitable for plasma miRNA quantification by qRT-PCR, as it primarily reflects ribosomal RNA quality, which is typically absent in cell-free plasma (Cheng et al., 2013, McDonald et al., 2011, El-Khoury et al., 2016, Garcia-Elias et al., 2017). This is further supported by Garcia-Elias et al. (Garcia-Elias et al., 2017), who showed that the absence of 18S/28S rRNA does not preclude reliable plasma miRNA profiling via microarray. In such cases, alternative strategies are required. We therefore ensured RNA quality for qRT-PCR through the use of a validated, miRNA-specific extraction protocol and monitored technical consistency using the exogenous spike-in control *cel-miR-39* (McDonald et al., 2011). This approach compensates for the limitations of traditional RNA integrity metrics in plasma-based miRNA analysis. Still, further validation using standardized reference controls and independent cohorts is needed to establish robust quality assurance in qRT-PCR–based miRNA diagnostics.

Although our candidate selection focused on the quadrant exhibiting ΔHO > 0 and ΔCPP < 0 based on its biological interpretability, we recognize that miRNAs downregulated in both groups or specifically in the CPP group may also harbor relevant biomarkers for pain chronification or resolution. These categories warrant future investigation in larger cohorts.

Future studies should incorporate neuropathic pain cohorts and validate findings in larger, independent datasets. Longitudinal designs will help determine whether miRNA expression tracks with pain recurrence or treatment response over time. Moreover, multi-miRNA panels may provide superior diagnostic accuracy compared to single markers (Bhowmick et al., 2018, Kerschan-Schindl et al., 2021). Integration of Artificial Intelligence (AI)-based modeling and multi-omic data could further refine classification frameworks and support clinical translation.

## Conclusion

This exploratory study suggests that circulating miRNAs—particularly *let-7a*, *miR-26a*, and *miR-16*—may differentiate nociceptive from nociplastic pain and track responses to surgical or psychological treatment. While preliminary, these findings highlight the potential of miRNA profiling as an objective, mechanism-based tool for pain diagnosis and personalization of therapy. Future validation in larger, mechanistically diverse cohorts is warranted to support clinical implementation.

Funding

This work was supported by the Japan Society for the Promotion of Science (JSPS) KAKENHI Grant Number 17 K09049 awarded to HN.

## Study pre-registration statement

This study was pre-registered in the University Hospital Medical Information Network (UMIN) Clinical Trials Registry (UMIN study ID: 000032416). The study protocol, including hypotheses and analysis plan, is available at https://rctportal.niph.go.jp/s/detail/um?trial_id=UMIN000032416.

## Declaration of Generative AI and AI-assisted technologies in the writing process

During the preparation of this work the authors used ChatGPT 4o in order to improve language and readability. After using this tool, the authors reviewed and edited the content as needed and take full responsibility for the content of the publication.

## CRediT authorship contribution statement

**Hiroyuki Nishie:** Writing – original draft, Project administration, Methodology, Investigation, Funding acquisition, Data curation. **Hideki Nakatsuka:** Writing – review & editing, Supervision, Project administration, Methodology, Investigation. **Kazunori Iwasa:** Writing – review & editing, Investigation, Formal analysis, Data curation. **Yuka Sakuta:** Writing – review & editing, Investigation, Formal analysis, Data curation. **Yuichiro Toda:** Writing – review & editing, Supervision, Investigation, Formal analysis, Data curation. **Shigeru Mitani:** Writing – review & editing, Supervision, Project administration, Investigation, Data curation. **Takeshi Nagasaka:** Writing – review & editing, Writing – original draft, Visualization, Validation, Supervision, Project administration, Methodology, Formal analysis, Data curation, Conceptualization.

## Declaration of competing interest

The authors declare that they have no known competing financial interests or personal relationships that could have appeared to influence the work reported in this paper.

## Data Availability

Data will be made available on request.
