# Supplementary material for: Circulating microRNAs differentiate nociceptive and nociplastic pain: An exploratory study
Source: Neurobiol Pain. 2025 Jul 5;18:100191. doi: 10.1016/j.ynpai.2025.100191 (PMC12275945; doi:10.1016/j.ynpai.2025.100191)
Supplement: Supplementary Data 1 [file mmc1.docx]

**Supplementary Files**

**Circulating microRNAs Differentiate Nociceptive and Nociplastic Pain: An Exploratory Study**

Hiroyuki Nishie, MD, Ph.D^1, 2^, Hideki Nakatsuka, MD, Ph.D^2^, Kazunori Iwasa, Ph.D^3^, Yuka Sakuta, MD, Ph.D^2^, Yuichiro Toda, MD, Ph.D^2^, Shigeru Mitani, MD, Ph.D^4^, and Takeshi Nagasaka, MD, Ph.D^1^

^1^Department of Advanced Oncology, Kawasaki Medical School, Kurashiki-City, Okayama, 701-0192, Japan.

^2^Department of Anesthesiology and Intensive Care Medicine, Kawasaki Medical School Hospital, Kurashiki-City, Okayama, 701-0192, Japan.

^3^Department of Psychology, Graduate School of Sustainable System Sciences, Osaka Metropolitan University, Osaka-city, Osaka, Japan.

^4^Department of Bone and Joint Surgery, Kawasaki Medical School Hospital, Kurashiki-City, Okayama, 701-0192, Japan.

**Supplementary Methods**

**Inclusion and Exclusion Criteria for Participants**

**The HO Group**: The HO group included patients scheduled for surgery for hip osteoarthritis. Patients were recruited during their preoperative outpatient visit, where they were informed about the study and provided consent.

Inclusion criteria:

(1) Patients scheduled for total hip arthroplasty with ASA1 (general status classification of one [ideal] or two by the American Society of Anesthesiologists).

(2) Patients aged 20–80 years.

(3) Patients who understood the purpose and content of the study and gave written consent to participate voluntarily.

Exclusion criteria:

(1) Patients with severe pain in a location other than the affected hip joint.

(2) Patients with severe cognitive dysfunction (e.g., delirium, dementia, intellectual disability).

(3) Patients with difficulty communicating, reading, and writing in Japanese.

(4) Patients with contraindications to magnetic resonance imaging (MRI) imaging (due to concurrent head MRI imaging studies).

(5) Patients deemed unsuitable for the study by the principal investigator.

**The CPP Group**: The CPP group consisted of patients with persistent pain who were scheduled for cognitive-behavioral therapy (CBT). Imaging and blood tests showed no pathological conditions explaining the pain.

Inclusion criteria:

(1) Patients with persisting pain for over three months.

(2) Patients aged 20–80 years.

(3) Patients who understood the study and provided written informed consent.

Exclusion criteria:

(1) Patients requiring immediate treatment for illnesses detected during pre-study evaluation.

(2) Patients with a history or current admission for alcohol or drug abuse.

(3) Patients with a history or current admission for manic or psychotic states.

(4) Patients with significant suicidal ideation.

(5) Patients with severe cognitive impairment.

(6) Patients with communication difficulties in Japanese.

(7) Patients with pain caused by injury or surgery.

(8) Patients seeking compensation or litigation for pain.

(9) Patients with a pain level of 10 out of 10.

(10) Patients with contraindications to MRI imaging.

(11) Patients deemed unsuitable by the principal investigator.

Patients underwent eight sessions of CBT over 12 ± 2 weeks with a clinical psychologist, based on the protocol by Hosogoshi et al (1). Evaluations were conducted three months after CBT completion (approximately six months after the start of CBT).

**The Control Group**

Inclusion criteria:

(1) No pain lasting more than three months.

(2) Aged 20–80 years.

(3) Provided written informed consent.

Exclusion criteria:

(1) Patients requiring immediate treatment for illnesses detected during pre-study evaluation.

(2) Patients with a history or current admission for alcohol or drug abuse.

(3) Patients with a history or current admission for manic or psychotic states.

(4) Patients with significant suicidal ideation.

(5) Patients with severe cognitive impairment.

(6) Patients with communication difficulties in Japanese.

(7) Patients with pain caused by injury or surgery.

(8) Patients seeking compensation or litigation for pain.

(9) Patients with a pain level of 10 out of 10.

(10) Patients with contraindications to MRI imaging.

(11) Patients deemed unsuitable by the principal investigator.

(12) an EQ-5D-5L score of less than 1

(13) a Numerical Rating Scale (NRS) score greater than 3 for maximum, minimum, average, or current pain levels over the past week**.**

**Biological Justification for miRNA Selection Thresholds**

The selection thresholds (Δ HO > 10, -100 < Δ CPP < 0, and HO/CPP ratio < 0.7) were set to identify miRNAs strongly associated with nociceptive pain resolution while minimizing the influence of nociplastic pain mechanisms. While no single study has directly validated these specific cutoffs, these thresholds were determined based on existing literature on miRNA involvement in inflammation, neuroplasticity, and chronic pain modulation.

**1. Δ HO > 10: Selecting miRNAs Highly Upregulated in the HO Group**

This threshold prioritized miRNAs with substantial upregulation in response to tissue injury resolution following total hip arthroplasty (THA). Studies have indicated that inflammation-regulating miRNAs, such as *miR-126* and the *let-7* family, play key roles in vascular remodeling, inflammatory resolution, and tissue repair following injury (2, 3). While a universal threshold for biologically significant upregulation is not established, prior studies on post-injury inflammation and vascular healing suggest that certain miRNAs exhibit significant upregulation (>10-fold) during tissue recovery.

**2. -100 < Δ CPP < 0: Excluding miRNAs Upregulated in the CPP Group**

This threshold was set to exclude miRNAs highly upregulated in chronic primary pain (CPP) states, which are more likely to be driven by central sensitization rather than peripheral inflammation. miRNAs such as *miR-21* and *miR-146a*, known regulators of neuroinflammation, have been reported to exhibit dynamic changes in chronic pain conditions, often showing moderate downregulation (-10% to -50%) in some models of persistent pain (4). While Zeboudj et al. demonstrated that silencing *miR-21* reverses neuropathic allodynia, its role in TGF-β-related macrophage activation suggests a broader relevance to chronic and nociplastic pain modulation (4).

**3. HO/CPP Preoperative Ratio < 0.7: Ensuring Baseline Specificity for Nociceptive Pain**

This threshold was set to exclude miRNAs that are already highly expressed in nociplastic pain patients prior to treatment, as these may reflect persistent neuroplastic adaptations rather than nociceptive pain mechanisms. Some nociplastic pain-associated miRNAs, such as *miR-26a* and *miR-103a*, are elevated prior to treatment as a result of prolonged neuroinflammation and maladaptive plasticity, potentially contributing to heightened central pain perception. While more research is needed to determine precise preoperative expression patterns of these miRNAs, this threshold ensures that chronic pain mechanisms do not confound nociceptive pain-specific biomarkers.

Although these selection criteria are based on biological plausibility and existing literature, large-scale validation studies are required to confirm their accuracy in distinguishing nociceptive from nociplastic pain. Further studies should investigate how these miRNAs interact with established pain pathways, particularly in relation to central sensitization and immune-neural signaling.

**References**

1. Hosogoshi H, Iwasa K, Fukumori T, Takagishi Y, Takebayashi Y, Adachi T, et al. Pilot study of a basic individualized cognitive behavioral therapy program for chronic pain in Japan. Biopsychosoc Med. 2020;14:6.

2. Harris TA, Yamakuchi M, Ferlito M, Mendell JT, Lowenstein CJ. MicroRNA-126 regulates endothelial expression of vascular cell adhesion molecule 1. Proc Natl Acad Sci U S A. 2008;105(5):1516-21.

3. Pan J, Qu M, Li Y, Wang L, Zhang L, Wang Y, et al. MicroRNA-126-3p/-5p Overexpression Attenuates Blood-Brain Barrier Disruption in a Mouse Model of Middle Cerebral Artery Occlusion. Stroke. 2020;51(2):619-27.

4. Zeboudj L, Sideris-Lampretsas G, Silva R, Al-Mudaris S, Picco F, Fox S, et al. Silencing miR-21-5p in sensory neurons reverses neuropathic allodynia via activation of TGF-beta-related pathway in macrophages. J Clin Invest. 2023;133(11).

**
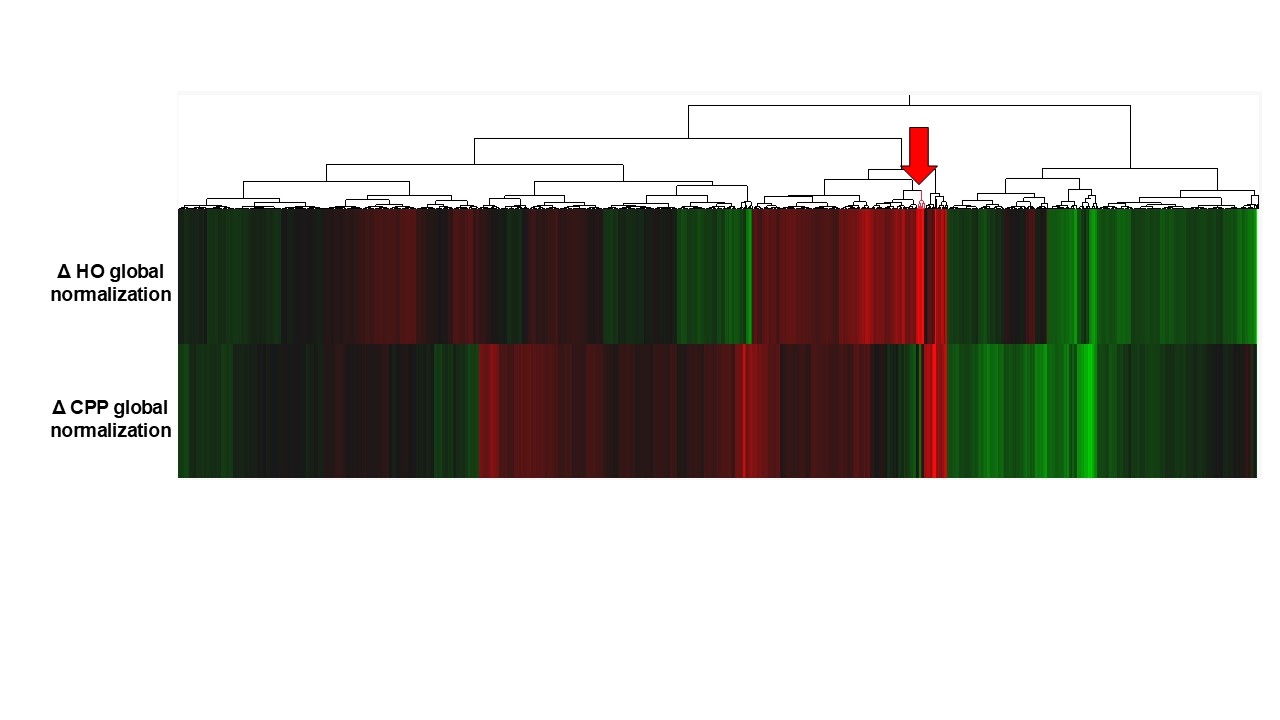
Supplementary Figure 1. A heat map derived from a cluster analysis.**

The red arrow indicates a cluster that exhibited an increased Δ HO global normalization while showing either a decreased or unchanged Δ CPP global normalization.

**Supplementary Table 2. Characteristics of Participants.**

|  | **CPP (N=11)** | **HO (N=13)** | **Control (N=7)** |
| --- | --- | --- | --- |
| Age (years), Median [min, max] | 55 [24, 74] | 63 [56, 78] | 41 [28, 54] |
| Sex, No (%) |  |  |  |
| Female | 6 (54.5) | 13 (100) | 7(100) |
| Male | 5 (45.5) | 0 (0) | 0 (0) |
| Weight (kg), Median [min, max] | 55.0 [38.0, 90.0] | 59.3 [41.9, 68.7] | 51.3 [46.1, 67.2] |
| Height (cm), Median [min, max] | 155.1 [148.0, 174.4] | 153.1 [139.8, 168.7] | 160.5 [148.6, 162.3] |
| Body Mass Index (kg/m2), Median [min, max] | 24.5 [16.0, 31.9] | 24.85 [19.0, 29.8] | 20.2 [17.8, 26.8] |

**Supplementary Table 3. Site of pain and treatment outcome in CPP patients.**

| **Age (years)** | **Sex** | **Site of pain** | **CBT Outcome** |
| --- | --- | --- | --- |
| 72 | Female | Back pain, Low back pain, Buttocks pain, Leg pain | Complete |
| 55 | Male | Low back pain | Complete |
| 67 | Female | Right hip joint pain | Complete |
| 44 | Male | Low back pain | Drop out |
| 49 | Male | Whole body pain | Complete |
| 53 | Male | Whole body pain | Drop out |
| 41 | Female | Both shoulder pain | Complete |
| 55 | Female | Low back pain, Leg pain | Complete |
| 74 | Female | Low back pain, Leg pain, Genital pain | Drop out |
| 61 | Female | Left arm pain | Complete |
| 24 | Female | Whole body pain | Complete |

**Supplementary Table 4. Classification of 1,189 circulating miRNAs into a 2×2 matrix according to Δ global normalization in the HO (nociceptive) and CPP (nociplastic) groups.**

| **Category, no (% in total)** | **Δ CPP global normalization < 0** | **Δ CPP global normalization > 0** | **Total** |
| --- | --- | --- | --- |
| **Δ HO global normalization < 0** | 501 (42.1%) | 434 (36.5%) | 935 (78.6%) |
| **Δ HO global normalization > 0** | 84 (7.1%) | 170 (14.3%) | 254 (21.4%) |
| **Total** | 585 (49.2%) | 604 (50.8%) | 1189 (100) |

A total of 1,189 circulating miRNAs detected by microarray were classified into four quadrants based on the direction of expression change (Δ global normalization) in the HO (hip osteoarthritis; nociceptive pain) and CPP (chronic primary pain; nociplastic pain) groups following treatment.

The quadrants were defined as follows:

1. ΔHO > 0 / ΔCPP < 0: Upregulated in HO, downregulated in CPP (potential nociceptive-specific resolution markers)
2. ΔHO < 0 / ΔCPP < 0: Downregulated in both groups (common therapeutic responders)
3. ΔHO > 0 / ΔCPP > 0: Upregulated in both groups (non-specific treatment response)
4. ΔHO < 0 / ΔCPP > 0: Downregulated in HO, upregulated in CPP (potential nociplastic pain-related markers)

The table presents the number and proportion of miRNAs in each quadrant. This classification was a conceptual reference to support biomarker selection, as further illustrated in **Supplementary Table 6**.

**Supplementary Table 5. miRNA list derived from a cluster analysis.**

|  |  | **CPP** | **HO** |
| --- | --- | --- | --- |
| **miRNA** | **miRBase Accession Number** | **Δ CPP global normalization** | **Δ HO global normalization** |
| *hsa-miR-16-5p* | MIMAT0000069 | 0 | 1.07 |
| *hsa-let-7a-5p* | MIMAT0000062 | 0.46 | 1.39 |
| *hsa-let-7d-5p* | MIMAT0000065 | 0.29 | 1.29 |
| *hsa-miR-3680-3p* | MIMAT0018107 | -0.46 | 1.19 |
| *hsa-miR-22-5p* | MIMAT0004495 | -0.06 | 1.44 |
| *hsa-miR-526a-5p,*  *hsa-miR-520c-5p,*  *hsa-miR-518d-5p* | MIMAT0002845, MIMAT0005455,  MIMAT0005456 | 0.01 | 1.12 |
| *hsa-miR-7154-3p* | MIMAT0028219 | 0.26 | 1.24 |
| *hsa-miR-26b-5p* | MIMAT0000083 | 0.05 | 1.89 |

Δ global normalizations were evaluated by the mean post-treatment minus pre-treatment log2 ratio.

**Supplementary Table 6. Representative miRNAs by Quadrant.**

| **Category** | **miRNA** | **HO/CPP Pre Ratio** | **ΔHO (Post–Pre)** | **ΔCPP (Post–Pre)** | **Known/Potential Function** |
| --- | --- | --- | --- | --- | --- |
| **(A)** | hsa-miR-451a | 0.499 | 428.335 | -229.462 | Erythrocyte miRNA; sensitive to hemolysis; inflammation marker |
|  | hsa-miR-16-5p | 0.308 | 65.462 | -55.968 | Apoptosis, immune modulation, neuroinflammation |
|  | hsa-miR-6777-5p | 0.853 | 64.977 | -0.711 | Unknown function; potential regulatory role |
|  | hsa-miR-320e | 0.657 | 57.476 | -9.729 | Inflammation, stress response |
|  | hsa-let-7a-5p | 0.283 | 56.489 | -6.538 | Neuroinflammation, microglial regulation, ASK1 inhibition |
| **(B)** | hsa-miR-4506 | 0.823 | -0.027 | -0.766 | Not well defined |
|  | hsa-miR-5008-3p | 0.967 | -0.034 | -0.235 | Not well defined; possible immune association |
|  | hsa-miR-103a-1-5p | 0.727 | -0.036 | -0.622 | Glucose metabolism, neural plasticity |
|  | hsa-miR-2467-3p | 0.743 | -0.121 | -3.39 | Not well characterized |
|  | hsa-miR-4421 | 0.678 | -0.207 | -1.682 | Unknown; low expression miRNA |
| **(C)** | hsa-miR-296-3p | 0.772 | 52.554 | 53.723 | Not annotated |
|  | hsa-miR-6717-5p | 0.805 | 48.524 | 50.451 | Not annotated |
|  | hsa-miR-24-3p | 0.777 | 45.674 | 23.506 | Not annotated |
|  | hsa-miR-920 | 0.666 | 44.694 | 12.166 | Not annotated |
|  | hsa-miR-6822-5p | 0.876 | 37.673 | 0.743 | Not annotated |
| **(D)** | hsa-miR-518c-5p | 1.085 | -0.024 | 3.719 | Not annotated |
|  | hsa-miR-7843-5p | 1.03 | -0.025 | 0.173 | Not annotated |
|  | hsa-miR-3137 | 1.044 | -0.055 | 3.286 | Not annotated |
|  | hsa-miR-3622b-5p | 0.885 | -0.069 | 6.069 | Not annotated |
|  | hsa-miR-6823-5p | 0.874 | -0.091 | 7.22 | Not annotated |

This table presents representative circulating miRNAs categorized into each quadrant of a 2×2 matrix, based on their expression changes before and after treatment in both the HO (hip osteoarthritis; nociceptive pain) and CPP (chronic primary pain; nociplastic pain) groups. Each miRNA is annotated with its known or proposed biological role—such as involvement in neuroinflammation, immune signaling, or angiogenesis—based on published literature. Quadrants are labeled for clarity as follows:

(A) ΔHO > 0, ΔCPP < 0

(B) ΔHO < 0, ΔCPP < 0

(C) ΔHO > 0, ΔCPP > 0

(D) ΔHO < 0, ΔCPP > 0.

**Supplementary Table 7. Relative expression levels of Candidate miRNAs**

|  | **miRNA relative expression level, mean (95%CI)** | | | | | | | | | | | |  |
| --- | --- | --- | --- | --- | --- | --- | --- | --- | --- | --- | --- | --- | --- |
| **miRNA** | **Control (n=7)** | | **CPP** | | | |  | | **HO** | | | |  |
|  |  |  | **Pre-treatment (n=11)** | | **Post-treatment (n=8)** | |  | | **Pre-treatment (n=13)** | | **Post-treatment (n=11)** | |  |
| ***let-7a*** | | 0.42 (0.24 - 0.59) | | 0.45 (0.26 - 0.65) | | 0.39 (0.25 - 0.53) | |  | | 0.15 (0.06 - 0.25) | | 0.45 (0.34 - 0.57) | |
| ***let-7d*** | | 0.38 (0.21 - 0.55) | | 0.41 (0.24 - 0.57) | | 0.32 (0.17 - 0.48) | |  | | 0.35 (0.18 - 0.52) | | 0.47 (0.35 - 0.59) | |
| ***miR-16*** | | 1.20 (0.77 - 1.63) | | 1.17 (0.82 - 1.52) | | 0.84 (0.57 - 1.11) | |  | | 0.99 (0.53 - 1.45) | | 1.20 (0.88 - 1.53) | |
| ***miR-21*** | | 0.86 (0.56 - 1.16) | | 0.78 (0.53 - 1.03) | | 0.49 (0.28 - 0.69) | |  | | 1.20 (0.93 - 1.48) | | 0.85 (0.68 - 1.01) | |
| ***miR-26a*** | | 1.09 (0.54 - 1.64) | | 0.53 (0.41 - 0.65) | | 0.43 (0.16 - 0.71) | |  | | 0.51 (0.26 - 0.76) | | 0.64 (0.45 - 0.83) | |
| ***miR-103*** | | 0.39 (0.16 - 0.61) | | 0.45 (0.30 - 0.60) | | 0.36 (0.14 - 0.57) | |  | | 0.51 (0.17 - 0.86) | | 0.54 (0.38 - 0.71) | |
| ***miR-126*** | | 0.76 (0.42 - 1.11) | | 0.57 (0.44 - 0.70) | | 0.41 (0.18 - 0.64) | |  | | 0.91 (0.64 - 1.18) | | 0.58 (0.43 - 0.72) | |
| ***miR-146a*** | | 0.92 (0.55 - 1.29) | | 0.61 (0.53 - 0.70) | | 0.43 (0.22 - 0.64) | |  | | 1.02 (0.70 - 1.34) | | 0.69 (0.53 - 0.85) | |
| ***miR-223*** | | 0.79 (0.49 - 1.10) | | 0.65 (0.48 - 0.81) | | 0.47 (0.26 - 0.68) | |  | | 0.63 (0.40 - 0.86) | | 0.59 (0.41 - 0.77) | |

**Supplementary Table 8. The relationships between changes in miRNA expression levels and clinical variables.**

|  |  | **CPP** | | |  | **HO** | | |
| --- | --- | --- | --- | --- | --- | --- | --- | --- |
| **miRNA** | **by Variable** | **Spearman *ρ*** | ***P*-value** | **Adjusted *P*-value** |  | **Spearman *ρ*** | ***P*-value** | **Adjusted *P*-value** |
| **Δ *let-7a*** | Δ EQ-5D-5L | 0.071 | 0.867 | 0.947 |  | -0.127 | 0.709 | 0.951 |
|  | Δ NRS maximum | -0.108 | 0.798 | 0.943 |  | -0.096 | 0.778 | 0.951 |
|  | Δ NRS minimum | -0.292 | 0.483 | 0.801 |  | -0.442 | 0.173 | 0.791 |
|  | Δ NRS average | -0.277 | 0.506 | 0.801 |  | -0.241 | 0.476 | 0.951 |
|  | Δ NRS current | -0.025 | 0.954 | 0.972 |  | -0.194 | 0.569 | 0.951 |
|  | Δ PDAS | -0.671 | 0.069 | 0.712 |  | 0.237 | 0.482 | 0.951 |
|  | Δ PHQ-9 | 0.096 | 0.820 | 0.943 |  | -0.320 | 0.337 | 0.881 |
|  | Δ PCS overall | -0.371 | 0.365 | 0.724 |  | 0.109 | 0.750 | 0.951 |
|  | Δ PCS rumination | -0.512 | 0.194 | 0.712 |  | 0.155 | 0.649 | 0.951 |
|  | Δ PCS helplessness | -0.515 | 0.192 | 0.712 |  | 0.014 | 0.968 | 0.968 |
|  | Δ PCS magnification | -0.337 | 0.414 | 0.745 |  | 0.056 | 0.870 | 0.951 |
|  | Δ TSK-11 | -0.575 | 0.136 | 0.712 |  | 0.073 | 0.831 | 0.951 |
|  | Δ PSEQ | 0.500 | 0.207 | 0.712 |  | -0.201 | 0.555 | 0.951 |
| **Δ *let-7d*** | Δ EQ-5D-5L | -0.095 | 0.823 | 0.943 |  | -0.064 | 0.853 | 0.951 |
|  | Δ NRS maximum | 0.012 | 0.977 | 0.986 |  | -0.106 | 0.758 | 0.951 |
|  | Δ NRS minimum | -0.114 | 0.788 | 0.943 |  | **-0.645** | **0.032** | 0.401 |
|  | Δ NRS average | -0.036 | 0.932 | 0.972 |  | -0.352 | 0.289 | 0.871 |
|  | Δ NRS current | 0.135 | 0.750 | 0.943 |  | -0.369 | 0.265 | 0.860 |
|  | Δ PDAS | -0.431 | 0.286 | 0.712 |  | 0.384 | 0.244 | 0.860 |
|  | Δ PHQ-9 | 0.362 | 0.379 | 0.727 |  | -0.517 | 0.103 | 0.791 |
|  | Δ PCS overall | -0.240 | 0.568 | 0.852 |  | 0.136 | 0.689 | 0.951 |
|  | Δ PCS rumination | -0.439 | 0.276 | 0.712 |  | 0.301 | 0.369 | 0.894 |
|  | Δ PCS helplessness | -0.455 | 0.257 | 0.712 |  | -0.087 | 0.800 | 0.951 |
|  | Δ PCS magnification | -0.072 | 0.865 | 0.947 |  | 0.065 | 0.848 | 0.951 |
|  | Δ TSK-11 | -0.455 | 0.257 | 0.712 |  | -0.087 | 0.800 | 0.951 |
|  | Δ PSEQ | 0.381 | 0.352 | 0.724 |  | -0.214 | 0.527 | 0.951 |
| **Δ *miR-16*** | Δ EQ-5D-5L | -0.024 | 0.955 | 0.972 |  | -0.355 | 0.285 | 0.871 |
|  | Δ NRS maximum | 0.048 | 0.910 | 0.972 |  | -0.101 | 0.768 | 0.951 |
|  | Δ NRS minimum | -0.381 | 0.352 | 0.724 |  | -0.051 | 0.882 | 0.951 |
|  | Δ NRS average | 0.072 | 0.865 | 0.947 |  | 0.120 | 0.724 | 0.951 |
|  | Δ NRS current | 0.577 | 0.134 | 0.712 |  | 0.028 | 0.936 | 0.966 |
|  | Δ PDAS | -0.216 | 0.608 | 0.871 |  | 0.123 | 0.718 | 0.951 |
|  | Δ PHQ-9 | -0.060 | 0.887 | 0.961 |  | -0.325 | 0.330 | 0.881 |
|  | Δ PCS overall | -0.168 | 0.692 | 0.937 |  | 0.473 | 0.142 | 0.791 |
|  | Δ PCS rumination | -0.366 | 0.373 | 0.727 |  | 0.415 | 0.205 | 0.851 |
|  | Δ PCS helplessness | -0.383 | 0.349 | 0.724 |  | 0.338 | 0.310 | 0.881 |
|  | Δ PCS magnification | 0.157 | 0.711 | 0.943 |  | 0.112 | 0.743 | 0.951 |
|  | Δ TSK-11 | -0.527 | 0.180 | 0.712 |  | -0.073 | 0.831 | 0.951 |
|  | Δ PSEQ | 0.691 | 0.058 | 0.712 |  | -0.128 | 0.709 | 0.951 |
| **Δ *miR-21*** | Δ EQ-5D-5L | 0.119 | 0.779 | 0.943 |  | 0.082 | 0.811 | 0.951 |
|  | Δ NRS maximum | -0.217 | 0.606 | 0.871 |  | -0.404 | 0.218 | 0.851 |
|  | Δ NRS minimum | -0.228 | 0.587 | 0.869 |  | **-0.696** | **0.017** | 0.322 |
|  | Δ NRS average | -0.241 | 0.565 | 0.852 |  | -0.509 | 0.110 | 0.791 |
|  | Δ NRS current | 0.086 | 0.840 | 0.947 |  | -0.585 | 0.059 | 0.570 |
|  | Δ PDAS | -0.527 | 0.180 | 0.712 |  | 0.169 | 0.620 | 0.951 |
|  | Δ PHQ-9 | 0.096 | 0.820 | 0.943 |  | -0.339 | 0.308 | 0.881 |
|  | Δ PCS overall | -0.395 | 0.333 | 0.724 |  | 0.055 | 0.873 | 0.951 |
|  | Δ PCS rumination | -0.610 | 0.108 | 0.712 |  | 0.187 | 0.582 | 0.951 |
|  | Δ PCS helplessness | -0.611 | 0.108 | 0.712 |  | -0.192 | 0.572 | 0.951 |
|  | Δ PCS magnification | -0.241 | 0.565 | 0.852 |  | -0.145 | 0.671 | 0.951 |
|  | Δ TSK-11 | -0.599 | 0.117 | 0.712 |  | -0.183 | 0.591 | 0.951 |
|  | Δ PSEQ | 0.452 | 0.260 | 0.712 |  | -0.146 | 0.669 | 0.951 |
| **Δ *miR-26a*** | Δ EQ-5D-5L | 0.143 | 0.736 | 0.943 |  | 0.018 | 0.958 | 0.966 |
|  | Δ NRS maximum | -0.446 | 0.268 | 0.712 |  | -0.266 | 0.429 | 0.917 |
|  | Δ NRS minimum | -0.165 | 0.696 | 0.937 |  | **-0.678** | **0.022** | 0.322 |
|  | Δ NRS average | -0.374 | 0.362 | 0.724 |  | -0.463 | 0.152 | 0.791 |
|  | Δ NRS current | 0.135 | 0.750 | 0.943 |  | -0.498 | 0.119 | 0.791 |
|  | Δ PDAS | -0.347 | 0.399 | 0.742 |  | 0.434 | 0.183 | 0.791 |
|  | Δ PHQ-9 | -0.036 | 0.932 | 0.972 |  | -0.288 | 0.390 | 0.894 |
|  | Δ PCS overall | -0.467 | 0.243 | 0.712 |  | 0.164 | 0.631 | 0.951 |
|  | Δ PCS rumination | **-0.708** | **0.050** | 0.712 |  | 0.374 | 0.258 | 0.860 |
|  | Δ PCS helplessness | -0.683 | 0.062 | 0.712 |  | -0.096 | 0.779 | 0.951 |
|  | Δ PCS magnification | -0.277 | 0.506 | 0.801 |  | -0.019 | 0.957 | 0.966 |
|  | Δ TSK-11 | -0.599 | 0.117 | 0.712 |  | -0.183 | 0.591 | 0.951 |
|  | Δ PSEQ | 0.381 | 0.352 | 0.724 |  | -0.269 | 0.424 | 0.917 |
| **Δ *miR-103*** | Δ EQ-5D-5L | 0.071 | 0.867 | 0.947 |  | -0.018 | 0.958 | 0.966 |
|  | Δ NRS maximum | -0.108 | 0.798 | 0.943 |  | -0.193 | 0.570 | 0.951 |
|  | Δ NRS minimum | -0.292 | 0.483 | 0.801 |  | **-0.710** | **0.014** | 0.322 |
|  | Δ NRS average | -0.277 | 0.506 | 0.801 |  | -0.435 | 0.181 | 0.791 |
|  | Δ NRS current | -0.025 | 0.954 | 0.972 |  | -0.475 | 0.140 | 0.791 |
|  | Δ PDAS | -0.671 | 0.069 | 0.712 |  | 0.406 | 0.215 | 0.851 |
|  | Δ PHQ-9 | 0.096 | 0.820 | 0.943 |  | -0.476 | 0.139 | 0.791 |
|  | Δ PCS overall | -0.371 | 0.365 | 0.724 |  | 0.109 | 0.750 | 0.951 |
|  | Δ PCS rumination | -0.512 | 0.194 | 0.712 |  | 0.292 | 0.384 | 0.894 |
|  | Δ PCS helplessness | -0.515 | 0.192 | 0.712 |  | -0.132 | 0.698 | 0.951 |
|  | Δ PCS magnification | -0.337 | 0.414 | 0.745 |  | -0.019 | 0.957 | 0.966 |
|  | Δ TSK-11 | -0.575 | 0.136 | 0.712 |  | -0.050 | 0.883 | 0.951 |
|  | Δ PSEQ | 0.500 | 0.207 | 0.712 |  | -0.314 | 0.347 | 0.881 |
| **Δ *miR-126*** | Δ EQ-5D-5L | 0.143 | 0.736 | 0.943 |  | 0.191 | 0.574 | 0.951 |
|  | Δ NRS maximum | -0.446 | 0.268 | 0.712 |  | -0.450 | 0.165 | 0.791 |
|  | Δ NRS minimum | -0.165 | 0.696 | 0.937 |  | **-0.866** | **0.001** | 0.053 |
|  | Δ NRS average | -0.374 | 0.362 | 0.724 |  | **-0.639** | **0.034** | 0.401 |
|  | Δ NRS current | 0.135 | 0.750 | 0.943 |  | **-0.678** | **0.022** | 0.322 |
|  | Δ PDAS | -0.347 | 0.399 | 0.742 |  | 0.265 | 0.431 | 0.917 |
|  | Δ PHQ-9 | -0.036 | 0.932 | 0.972 |  | -0.375 | 0.255 | 0.860 |
|  | Δ PCS overall | -0.467 | 0.243 | 0.712 |  | -0.100 | 0.770 | 0.951 |
|  | Δ PCS rumination | -0.708 | 0.050 | 0.712 |  | 0.187 | 0.582 | 0.951 |
|  | Δ PCS helplessness | -0.683 | 0.062 | 0.712 |  | -0.388 | 0.238 | 0.860 |
|  | Δ PCS magnification | -0.277 | 0.506 | 0.801 |  | -0.257 | 0.445 | 0.930 |
|  | Δ TSK-11 | -0.599 | 0.117 | 0.712 |  | -0.101 | 0.769 | 0.951 |
|  | Δ PSEQ | 0.381 | 0.352 | 0.724 |  | -0.237 | 0.483 | 0.951 |
| **Δ *miR-146a*** | Δ EQ-5D-5L | 0.214 | 0.610 | 0.871 |  | 0.373 | 0.259 | 0.860 |
|  | Δ NRS maximum | -0.530 | 0.177 | 0.712 |  | -0.601 | 0.051 | 0.537 |
|  | Δ NRS minimum | -0.254 | 0.544 | 0.849 |  | **-0.853** | **0.001** | 0.053 |
|  | Δ NRS average | -0.482 | 0.227 | 0.712 |  | **-0.732** | **0.011** | 0.307 |
|  | Δ NRS current | 0.000 | 1.000 | 1.000 |  | **-0.816** | **0.002** | 0.086 |
|  | Δ PDAS | -0.419 | 0.301 | 0.724 |  | 0.091 | 0.789 | 0.951 |
|  | Δ PHQ-9 | -0.169 | 0.690 | 0.937 |  | -0.192 | 0.571 | 0.951 |
|  | Δ PCS overall | -0.467 | 0.243 | 0.712 |  | -0.209 | 0.537 | 0.951 |
|  | Δ PCS rumination | -0.659 | 0.076 | 0.712 |  | 0.023 | 0.947 | 0.966 |
|  | Δ PCS helplessness | -0.647 | 0.083 | 0.712 |  | -0.502 | 0.115 | 0.791 |
|  | Δ PCS magnification | -0.313 | 0.450 | 0.798 |  | -0.323 | 0.333 | 0.881 |
|  | Δ TSK-11 | -0.635 | 0.091 | 0.712 |  | -0.306 | 0.360 | 0.894 |
|  | Δ PSEQ | 0.476 | 0.233 | 0.712 |  | -0.059 | 0.863 | 0.951 |
| **Δ *miR-223*** | Δ EQ-5D-5L | 0.405 | 0.320 | 0.724 |  | -0.318 | 0.340 | 0.881 |
|  | Δ NRS maximum | -0.615 | 0.105 | 0.712 |  | 0.128 | 0.707 | 0.951 |
|  | Δ NRS minimum | -0.127 | 0.765 | 0.943 |  | -0.175 | 0.607 | 0.951 |
|  | Δ NRS average | -0.639 | 0.088 | 0.712 |  | 0.093 | 0.787 | 0.951 |
|  | Δ NRS current | -0.184 | 0.663 | 0.934 |  | 0.046 | 0.893 | 0.951 |
|  | Δ PDAS | -0.539 | 0.168 | 0.712 |  | 0.297 | 0.375 | 0.894 |
|  | Δ PHQ-9 | -0.289 | 0.487 | 0.801 |  | -0.503 | 0.114 | 0.791 |
|  | Δ PCS overall | -0.431 | 0.286 | 0.712 |  | 0.455 | 0.160 | 0.791 |
|  | Δ PCS rumination | -0.634 | 0.091 | 0.712 |  | 0.469 | 0.145 | 0.791 |
|  | Δ PCS helplessness | -0.575 | 0.136 | 0.712 |  | 0.279 | 0.407 | 0.916 |
|  | Δ PCS magnification | -0.530 | 0.177 | 0.712 |  | 0.351 | 0.291 | 0.871 |
|  | Δ TSK-11 | -0.479 | 0.230 | 0.712 |  | -0.224 | 0.508 | 0.951 |
|  | Δ PSEQ | 0.286 | 0.493 | 0.801 |  | 0.046 | 0.894 | 0.951 |

Δ denotes the difference between the relative expression of miRNA or the questionnaire survey scores before and after treatment, calculated by subtracting the pre-treatment value from the post-treatment value.

Spearman’s rank correlation coefficient (*ρ*) was calculated, and *P*-values were evaluated using a permutation test.

Adjusted *P*-values were evaluated using the Benjamini-Hochberg method.

**Supplementary Table 9. Previously reported associations between *let-7a*, *miR-26a*, and *miR-16* and chronic pain conditions.**

| **miRNA** | **Associated Pain Conditions** | **Reference** |
| --- | --- | --- |
| *let-7a* | Neuropathic pain, osteoarthritis | (5, 6) |
| *miR-26a* | Neuroinflammation, central sensitization | (7) |
| *miR-16* | Fibromyalgia, chronic widespread pain | (8) |

**Reference**

5. Fiebich BL, Batista CRA, Saliba SW, Yousif NM, de Oliveira ACP. Role of Microglia TLRs in Neurodegeneration. Front Cell Neurosci. 2018;12:329.

6. Ma Y, Shen N, Wicha MS, Luo M. The Roles of the Let-7 Family of MicroRNAs in the Regulation of Cancer Stemness. Cells. 2021;10(9).

7. Kumar A, Bhatia HS, de Oliveira AC, Fiebich BL. microRNA-26a modulates inflammatory response induced by toll-like receptor 4 stimulation in microglia. J Neurochem. 2015;135(6):1189-202.

8. Polli A, Godderis L, Ghosh M, Ickmans K, Nijs J. Epigenetic and miRNA Expression Changes in People with Pain: A Systematic Review. J Pain. 2020;21(7-8):763-80.
